# Feasibility of continuous high-resolution bioreactance monitoring during cesarean delivery under spinal anesthesia

**DOI:** 10.3389/fmed.2025.1647102

**Published:** 2025-10-30

**Authors:** Christine Gaik, Tabea Bahle, Corinna Keil, Hinnerk Wulf, Benjamin Vojnar

**Affiliations:** ^1^Philipps University Marburg, Marburg, Germany; ^2^Department of Anesthesiology and Intensive Care Medicine, University Hospital Giessen and Marburg, Marburg, Germany; ^3^Department of Obstetrics and Perinatal Medicine, University Hospital of Giessen and Marburg, Marburg, Germany

**Keywords:** bioreactance technology, cesarean delivery, hypotension, spinal anesthesia, stroke volume index

## Abstract

**Purpose:**

This study is the first to apply bioreactance-based hemodynamic monitoring with a 4-s interval during cesarean delivery under spinal anesthesia. We evaluated the feasibility of continuous, high-resolution perioperative monitoring in a routine clinical setting, with a particular focus on the temporal relationship between hypotension and changes in advanced hemodynamic parameters.

**Methods:**

This prospective observational study conducted between December 2023 and April 2024 included 51 healthy parturients scheduled for elective cesarean delivery under spinal anesthesia were included. All participants underwent continuous non-invasive hemodynamic monitoring using bioreactance technology. Hemodynamic parameters were recorded at 4-s intervals. The primary outcome was the relative change in the stroke volume index (SVI) from baseline. The secondary endpoints included signal quality, data integrity, and trends in hemodynamic parameters.

**Results:**

A reduction in the stroke volume index (SVI) of ≥20% was observed in 29 of 51 patients. The median percentage change in SVI from baseline was −18.9% [IQR − 31.5 to −6.1]; *p* < 0.001. The median time from the onset of relevant SVI decline to the occurrence of hypotension was 2:20 min [IQR 1:16–3:56]. The total cumulative observation time for all patients was 3,781 min. At a 4-s sampling interval, approximately 56,713 data points per hemodynamic parameter were expected. The signal loss was minimal, with less than 0.5% missing data per parameter.

**Conclusion:**

This method proved to be feasible and yielded stable, high-resolution hemodynamic data. Among all parameters, SVI showed the most consistent baseline values prior to anesthesia. It also demonstrated the most pronounced change, with a statistically significant decline in the majority of subjects between spinal anesthesia and the onset of first hypotension. In such cases, the marked decline in SVI may serve as an early indicator of impending hemodynamic compromise. These results from a low-risk obstetric cohort may inform future research on high-risk populations.

## Introduction

Single-shot spinal anesthesia (SPA) is the most commonly used anesthetic technique for cesarean delivery (CD) (1). Depending on the definition of hypotension, up to 74% of women undergoing CD under SPA experience significant hypotension (2, 3). Hypotension has adverse effects on both the mother and newborn, especially if it is not treated or prevented early (3, 4). Therefore, hypotension must first be clearly identified. Oscillometric non-invasive blood pressure (NIBP) measurement is commonly used to monitor blood pressure (BP) during CD, typically with short measurement intervals of 1–2.5 min (3). Alternative technologies, such as finger cuff systems, have been investigated as potential methods for enabling non-invasive, continuous BP measurement during CD (5–8). Although these technologies are promising, they may have certain limitations in routine obstetric anesthesia practice. The placement of a finger cuff and forearm-mounted transmitter unit, which relays the signal to the monitor via a connecting cable, may be perceived as uncomfortable by some patients, particularly postpartum, when arm mobility is required to hold the newborn. Additionally, the relatively high cost of a single-use finger cuff may limit its broader clinical applicability.

In addition to established techniques for advanced hemodynamic monitoring, the bioreactance (BR) method may offer a non-invasive, continuous and patient-friendly alternative. BR-based CO monitoring analyzes the phase shifts of a high-frequency electrical current across the thorax (9). Four dual-surface electrodes were applied bilaterally to the upper and lower thorax, with two positioned above and two below the heart, respectively. The system detects phase shifts caused by the pulsatile aortic flow to calculate SV and other hemodynamic variables (10–12). Adhesive sensors can be applied quickly, and because the arms and hands remain unobstructed, vascular access and maternal–infant bonding are not compromised immediately after delivery. BR monitoring has already been validated against continuous, invasive thermodilution methods in intensive care and perioperative settings (9, 13–15), and has shown good agreement with echocardiography for measuring CO in pregnant women (10, 11). However, its application in obstetric anesthesia remains limited. Potential indications for advanced hemodynamic monitoring during CD under SPA include obstetric conditions associated with increased cardiovascular risk, such as severe preeclampsia, placenta accreta spectrum disorders, and anticipated major obstetric hemorrhage. However, such high-risk scenarios are relatively uncommon within the broader population of parturients undergoing cesarean delivery, and emergent settings occur in many cases. As a result, prospective investigations in these cohorts are scarce, and the clinical performance of novel monitoring technologies, such as bioreactance-based systems, has not been systematically evaluated in this context.

Therefore, we conducted a prospective observational study using the Starling Fluid Management Monitoring System (Baxter Healthcare, Deerfield, IL, USA) to assess the feasibility of bioreactance-based hemodynamic monitoring during CD under SPA. The primary aim was to determine whether this non-invasive technology enables continuous, high-resolution, and physiologically consistent measurements of key hemodynamic parameters. In addition to evaluating signal quality and data completeness, particular emphasis was placed on the dynamics of the SVI and its association with the onset of neuraxial anesthesia-induced hypotension. This approach provides a structured framework for characterizing hemodynamic trends during routine obstetric anesthesia and may inform future applications of bioreactance monitoring in high-risk obstetric populations.

## Materials and methods

### Study design and setting

This prospective observational study was registered in the German Clinical Trials Register (DRKS ID: DRKS00033174), approved by the Ethics Committee of Philipps University Marburg on July 28, 2023 (reference: 23-150 BO), and conducted at the Department of Anesthesiology and Intensive Care Medicine, Marburg University Hospital, Germany, between December 2023 and April 2024. Written informed consent was obtained from all patients prior to their inclusion in the study. This manuscript complies with the current “Strengthening the Reporting of Observational Studies in Epidemiology” (STROBE) guidelines.

### Inclusion and exclusion criteria

Women aged 18 years and older were eligible for inclusion if they were scheduled for elective CD under SPA. Exclusion criteria included patients younger than 18 years, those who declined SPA and required general anesthesia (GA), those converted to GA following SPA, and those with contraindications to BR monitoring. Contraindications included clinically relevant aortic valve insufficiency, severe anatomical abnormalities of the thoracic aorta, and use of external or internal pacemakers with unipolar electrodes.

### Preoperative assessment on the ward

In all included patients, BP, heart rate (HR), peripheral oxygen saturation (SpO_2_), and advanced hemodynamic parameters were measured on the day of CD in the ward using the BR monitor. All measurements were conducted in the supine position, following a 5-min resting period. An appropriately sized cuff was selected for oscillometric BP measurement based on the patient’s upper arm circumference, and the measurement intervals were set at 2 to 3 min.

### Anesthetic procedure and intraoperative data collection

All procedures were conducted in accordance with the clinical practice of our department and the current national guidelines on obstetric analgesia and anesthesia (16). These procedures were also aligned with the standards of other institutions and were consistent with the methodologies applied in comparable published studies (17, 18). Additionally, a study team consisting of one or two members was present to manage the documentation and operation of the BR monitor. After standard monitoring was established (SpO_2_, ECG, NIBP), patients were connected to the BR monitor, and advanced hemodynamic parameters were recorded automatically and continuously at 4-s intervals.

At least three BP measurements were taken prior to subcutaneous local anesthetic (LA) administration. SPA was performed with hyperbaric bupivacaine 0.5%. In line with departmental standards, each patient received a pressure-infused 500 mL crystalloid solution (co-loading) during SPA placement. Following SPA administration, the oscillometric BP measurement interval was set to 1 min and maintained until delivery and umbilical cord clamping. Hypotension was treated once it had occurred. Importantly, the BR monitor display was blinded to the clinical team, and the recorded values were used exclusively for study purposes and not for clinical decision-making.

### Definition of hypotension and baseline

Baseline systolic blood pressure (SBP) was defined as the mean of three consecutive SBP measurements obtained at rest prior to the administration of LA for SPA. Hypotension was defined as a decrease in SBP to less than 80% of the baseline, consistent with the most commonly used definitions in obstetric anesthesia literature (2). In cases where the subsequent oscillometric blood pressure measurement was not available, the occurrence of nausea or vomiting between the intrathecal administration of LA and delivery was recorded as a surrogate indicator of hypotension.

### Management of intraoperative hypotension

No treatment protocol was predefined; patient BP optimization followed in accordance with the clinical practice of our department and current national guidelines on obstetric analgesia and anesthesia (16). Internationally, phenylephrine and norepinephrine are the most commonly used for this purpose (19). In contrast, in German-speaking countries, the fixed-dose combination of cafedrine/theodrenaline (C/T, Akrinor™, Ratiopharm, Ulm, Germany), is a well-established treatment for hypotension in obstetric anesthesia (16, 20, 21). All patients received C/T, as intravenous boluses to restore MAP. Each ampoule contains 200 mg cafedrine and 10 mg theodrenaline. Cafedrine is a synthetic compound formed by covalent bonding of norephedrine and the xanthine derivative theophylline; theodrenaline results from linking theophylline to norepinephrine (22–24). The increase in BP is primarily initiated by alpha-adrenergic vasoconstriction mediated by the norepinephrine component of theodrenaline. This is followed by beta-1-adrenoceptor-mediated inotropic stimulation, induced by both norephedrine (from cafedrine) and norepinephrine (from theodrenaline). Theophylline, a phosphodiesterase inhibitor, potentiates this inotropic response by increasing the intracellular cAMP levels. Clinical data confirm a rapid onset, preload recruitment, and increased inotropy as the main mechanisms of action, with minimal effect on HR and only moderate vasoconstriction (22, 23, 25, 26). The dosage was reported only in terms of cafedrine content only.

### Statistical analysis and data management

#### Data documentation

All data from standard patient monitoring were transmitted via a wired connection to the BR monitor and subsequently exported as a standardized dataset for analysis. Data were downloaded onto a USB storage device at the end of the surgery. All relevant intraoperative interventions (e.g., time of SPA puncture, administration of fluids or medications, changes in patient positioning, skin incision, uterotomy, umbilical cord clamping, estimated blood loss, urine output, Apgar scores, umbilical arterial pH, and arterial base excess) were noted by the study team using a separate documentation sheet. For time synchronization, only the internal clock of the BR monitor was used.

#### Temporal aggregation of raw data to generate 20-s intervals

Many advanced hemodynamic monitoring devices report measurements aggregated over 20-s intervals (27). To evaluate the clinical relevance of the 4-s resolution used in this study, 20-s intervals were simulated by grouping the raw 4-s data into consecutive non-overlapping five-point blocks. Each block contained five sequential values of a given hemodynamic parameter, representing a 20-s period, from which an arithmetic mean was calculated. This approach enables a direct comparison between the original and aggregated measurement strategies.

#### Quantification of signal progression

To quantify the extent to which the temporally smoothed 20-s signal diverged from the original high-resolution 4-s data, two established error metrics from time-series analysis were applied: the mean absolute percentage error (MAPE) and root mean squared error (RMSE). Both are widely used in biomedical signal processing to evaluate the deviation between raw and smoothed signals, MAPE to capture relative deviation in percentage terms, and RMSE to quantifying absolute deviation in the original measurement units. RMSE was calculated as the square root of the average of the squared differences between each 4-s value (xᵢ), the corresponding 20-s block mean (x̄), n = number of values per block (in this case, 5), and *Σ* denotes the summation over all values in the block. Formally, this is expressed as: RMSE = √[(1 / n) × Σ (xᵢ − x̄)^2^]. The MAPE was computed as the average absolute percentage deviation of each 4-s value from the block mean, standardized by the magnitude of the individual values. This is expressed as: MAPE = (100 / n) × Σ |(xᵢ − x̄) / xᵢ|.

Lower RMSE and MAPE values indicate a close match between the smoothed and original signals, suggesting that the clinical information is preserved. In contrast, higher values may reflect a loss of temporal resolution that could obscure dynamic changes relevant to clinical decision-making.

### Statistical analysis

Descriptive analyses were conducted to summarize the relative changes in hemodynamic parameters before and after SPA. The distribution of continuous variables was assessed using the Shapiro–Wilk test. Because the data were not normally distributed, paired comparisons were performed using the Wilcoxon signed-rank test. Results were reported as median differences with corresponding interquartile ranges (IQRs). Statistical significance was set at a two-sided *p*-value <0.05. All statistical analyses were performed using SPSS Statistics version 30.0 (IBM Corp., Armonk, NY, USA) and Microsoft Excel version 16.92 (Microsoft Corp., Redmond, WA, USA).

## Results

**Figure 1:**
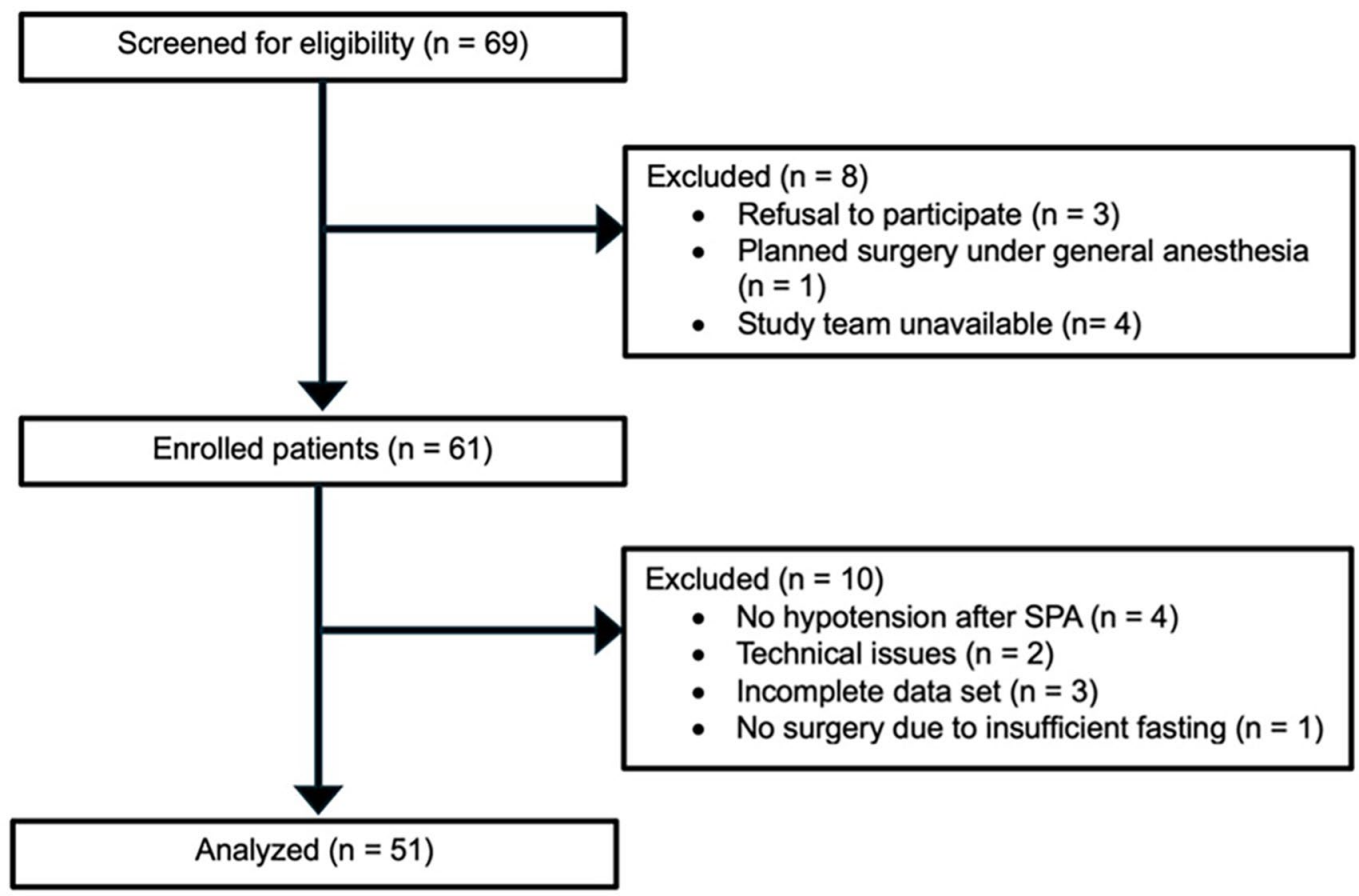
Study flow chart.

Figure 1 shows the CONSORT flow diagram for the present study. Sixty-nine pregnant women were screened for eligibility between December 2023 and April 2024. Eight patients were excluded prior to enrollment (three declined to participate, one had a planned CD under general anesthesia, and the study team was unavailable in four cases). A total of 61 patients were enrolled in this study. However, 10 patients were subsequently excluded from the analysis: four did not develop hypotension before the predefined end of the observation period (umbilical cord clamping), two were excluded due to technical issues, three had incomplete data sets, and one did not undergo surgery due to inadequate fasting. Thus, a total of 51 patients were included in the final analysis. All 51 patients underwent uncomplicated CD under SPA. The patient characteristics and anesthesia details are summarized in Table 1.

**Table 1:** Characteristics of 51 pregnant women scheduled for elective CD.

Variable	Value [median (IQR) or n (%)]
Age (years), median (IQR)	33 [30–37]
Height (cm)	166 (163–172)
BMI (kg m^−2^)	31 (28–37)
Puncture level (L), *n*	
L 2/3	22
L 3/4	27
L 4/5	2
Bupivacaine hyperbar (mg)	12.5 [12.5–13.0]
Sensory block level (Th), *n*	
Th 5	18
Th 5/6	18
Th 6	8
Th 6/7	4
Other, n/a	3
Time SPA* to first hypotension (s)	292 [204–368]
Time SPA* to delivery (min)	35 [32–41]
Surgery time (min)	71 [62–85]
Incidence of IONV, *n* (%) before delivery	23 (45)
Incidence of IONV, *n* (%) after delivery	11 (22)
Treatment of hypotension:	
C/T, *n* (%)	51 (100)
C/T (mg) before delivery	150 [100–200]
C/T (mg) in summary	160 [120–200]
Medication after delivery (total amount):	
Oxytocin (IE) - (infusion and bolus injections)	9 [7–11]
Esketamine (mg), *n* = 2	17.5 [16.25–18.75]
Fentanyl (mg), *n* = 14	0.05 [0.05–0.15]
Dexamethasone (mg), *n* = 31	4 [4–4]
Granisetrone (mg), *n* = 44	1 [1–1]
Other medication, n	7
Neonatal outcomes (*n* = 51):	
Apgar 0 min	9 [9–9]
Apgar 5 min	10 [10–10]
Apgar 10 min	10 [10–10]
Umbilical arterial pH	7,29 [7,26 – 7,33]
Umbilical arterial BE	–1 [–2 – –0,1]
Estimated blood loss (mL)	300 [300–500]
Estimated urine volume (mL)	200 [125–400]
Crystalloid infusion volume (mL)	1700 [1473–1875]

Values are median (IQR[range]) or number (proportion) unless otherwise stated. ASA = American Society of Anesthesiologists classification, BMI = Body mass index, C/T = cafedrine/theodrenaline (Akrinor), IONV = intraoperative nausea and vomiting, L = lumbar, SPA = spinal anesthesia, Th = thoracic. * Intrathecal administration of the LA.

### On ward

In the ward, prior to surgery, the median SBP was 124 mmHg [IQR 118–143], the MAP was 97 mmHg [IQR 88–107], the median SVI was 42 mL m^−2^ [IQR 38–47] and the median HR was 87 bpm [IQR 82–94]. Additional hemodynamic parameters recorded during the preoperative assessment are presented in Table 2.

**Table 2:** Average hemodynamic data at the defined time points.

Parameter	On ward	In the OR	1st hypotension	Percentage change from baseline to hypotension	*P*-value
HR (bpm)	87 [82–94]	98 [90–105]	100 [89–118]	+7.9 [−9.3 – 21.1] %	*p* = 0.042
CI (l min^−1^ m^−2^)	3.7 [3.5–4.0]	4.0 [3.6–4.6]	3.5 [3.1–4.1]	−11.9 [−28.8 – –0.9] %	*p* < 0.001
TPRI (dyn s ^−5^ m^−2^)	2,183 [1901–2,472]	1973 [1695–2,372]	1728 [1410–2,151]	−14.5 [−28.6 – 3.7] %	*p* = 0.005
SVI (ml m^−2^)	42 [38–47]	42 [37–48]	35 [28–40]	−18.9 [−31.5 – –6.1] %	*p* < 0.001
SBP (mmHg)	124 [118–143]	138 [129–152]	108 [100–119]	−23.0 [−27.9 – –20.6] %	*p* < 0.001
MAP (mmHg)	97 [88–107]	102 [93–112]	79 [71–84]	−26.0 [−32.2 – –19.6] %	*p* < 0.001
CPI (W m^−2^)	0.8 [0.7–0.9]	0.9 [0.8–1.0]	0.6 [0.5–0.7]	−34.2 [−46.4 – –20.7] %	*p* < 0.001

All ward measurements were obtained in the supine position after a 5-min resting period. OR values represent baseline measurements immediately prior to the administration of SPA. Percentage changes from baseline to first hypotension were calculated. Data are reported as median values with interquartile range (IQR). Reported *p*-values reflect the statistical significance of paired within-subject comparisons between baseline and 1st hypotension. CI = cardiac index, CPI = cardiac power index, HR = heart rate, MAP = mean arterial pressure, SBP = systolic blood pressure, SPA = spinal anesthesia, SVI = stroke volume index, TPRI = total peripheral resistance index.

### In the operating room

The median time from arrival in the delivery room to the administration of subcutaneous LA prior to SPA was 10 min [IQR 9–13]. During this pre-anesthetic phase, the median SBP was 138 mmHg [IQR 129–152], median MAP was 102 mmHg [IQR 93–112], median SVI was 42 mL m^−2^ [IQR 37–48] and median HR was 98 bpm [IQR 90–105]. Additional hemodynamic parameters measured in the operating room (OR) prior to SPA are detailed in Table 2.

### 1st hypotension

**Figure 2:**
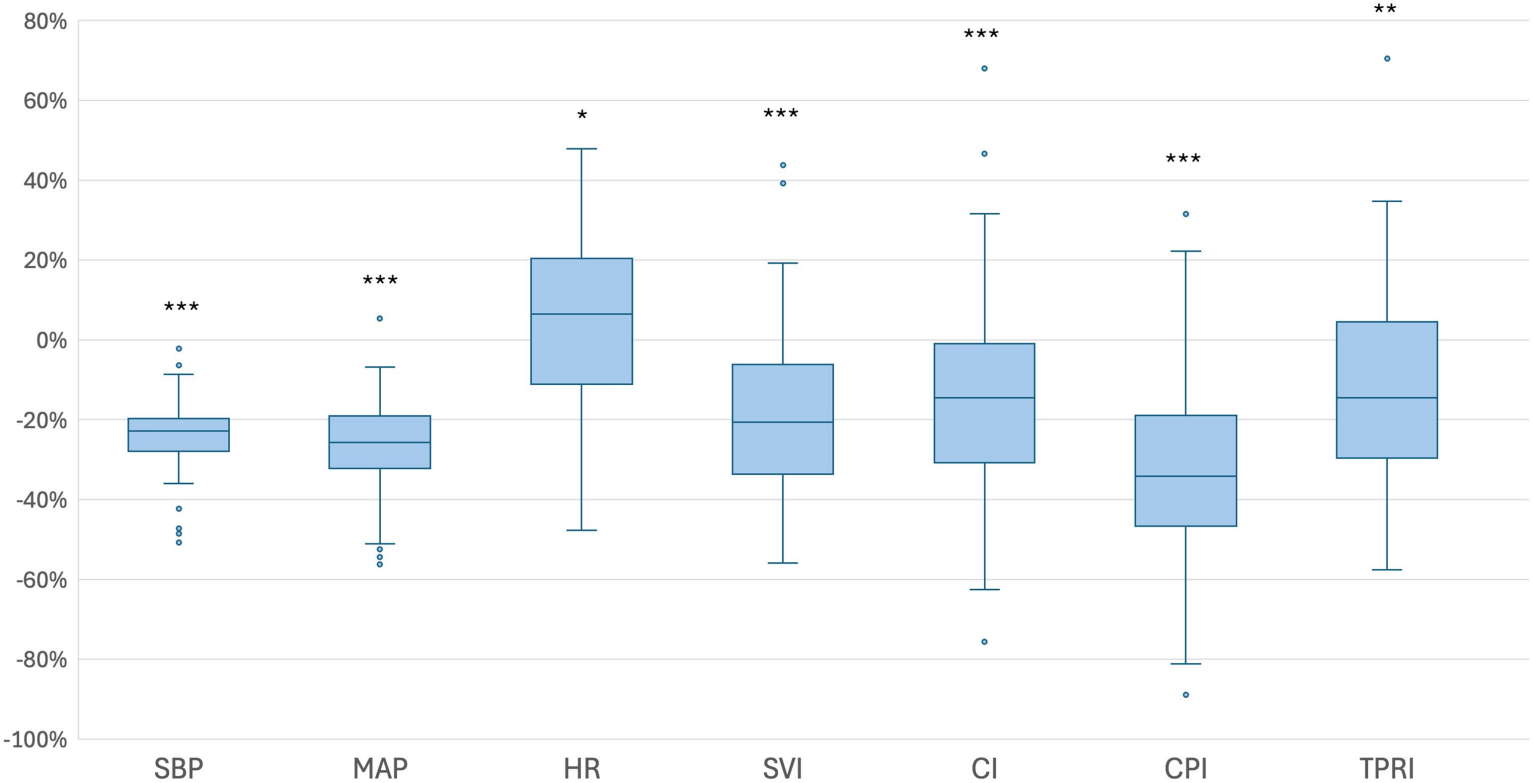
Boxplots illustrating the percentage changes in hemodynamic parameters from baseline to the first episode of hypotension. Baseline values were defined as the mean measurements obtained from arrival in the OR until the administration of subcutaneous LA. Boxes represent the IQR (25th to 75th percentile), horizontal lines indicate the medians, and whiskers extend to 1.5 times the IQR. Outliers beyond this range are displayed as individual dots. CI = cardiac index, CPI = cardiac power index, HR = heart rate, MAP = mean arterial pressure, SBP = systolic blood pressure, SVI = stroke volume index, TPRI = total peripheral resistance index, *p < 0.05, **p < 0.01, ***p < 0.001.

**Figure 3:**
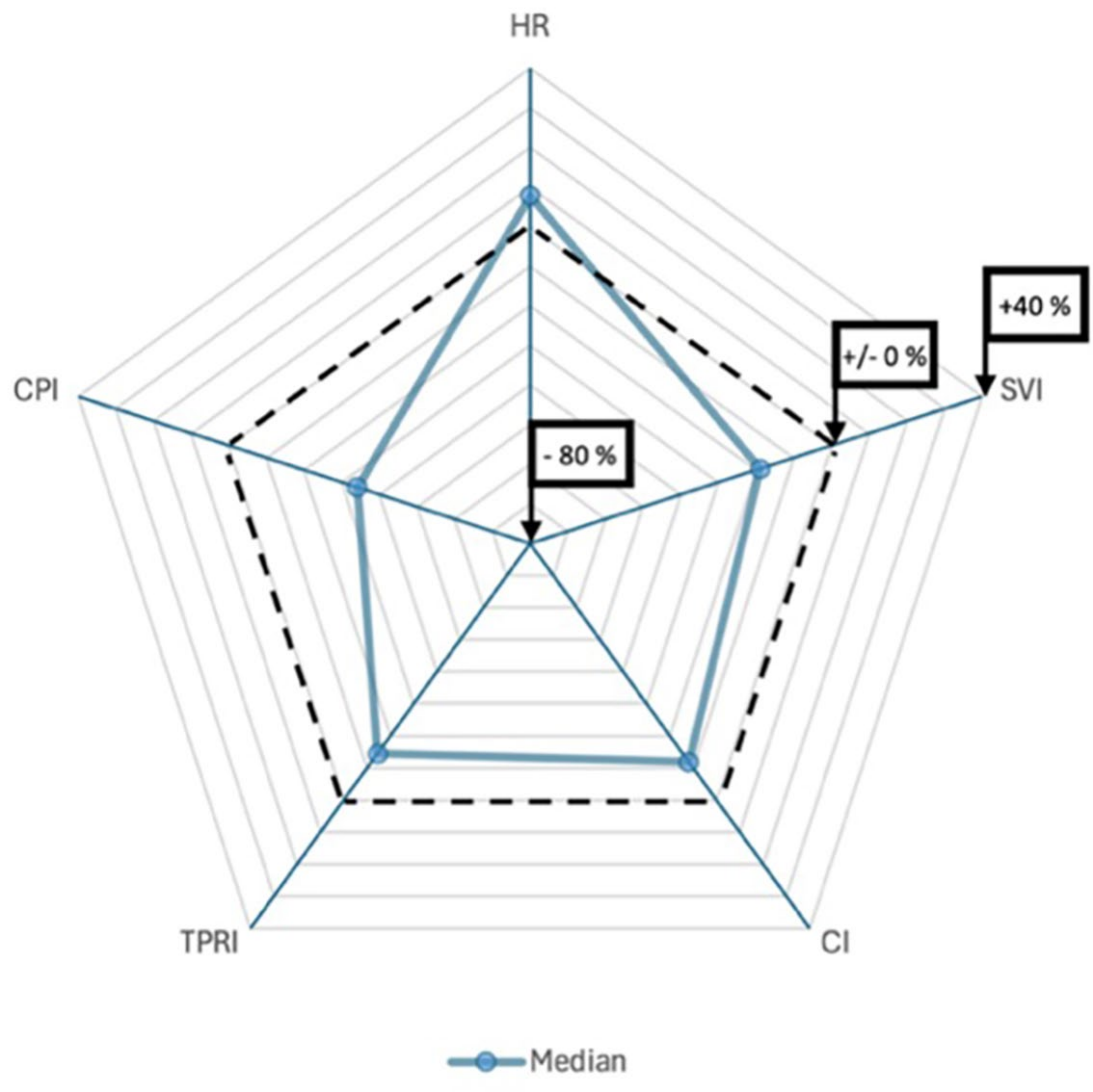
Spider plots showing the percentage changes from baseline to the time of first hypotension. Baseline values represent the mean measurements obtained from arrival in the OR until the administration of subcutaneous LA. The median change is indicated by a highlighted line, and the dotted line marks a change of ±0%. CI = cardiac index, CPI = cardiac power index, HR = heart rate, SVI = stroke volume index, TPRI = total peripheral resistance index.

**Figure 4:**
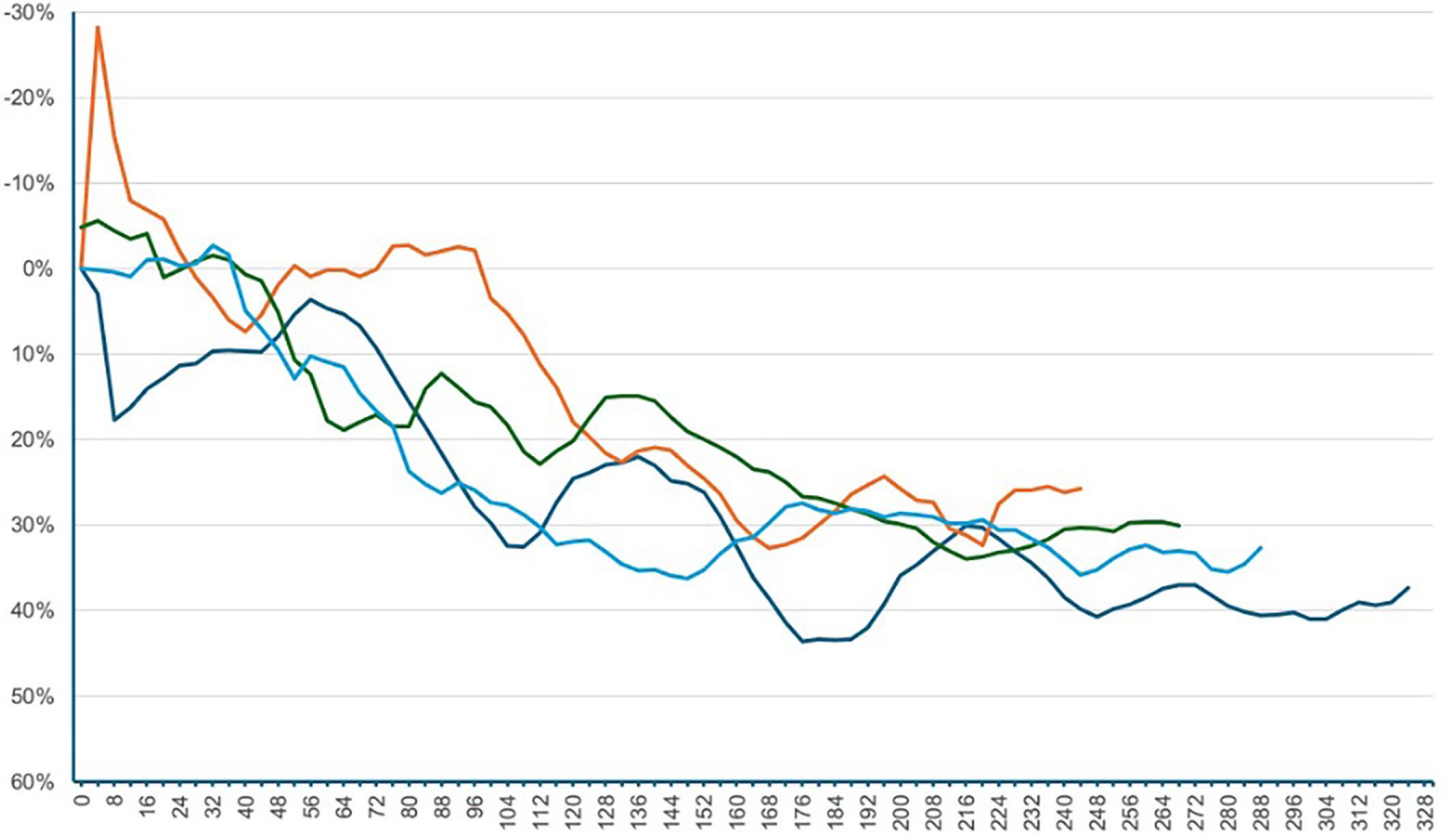
Dynamic changes in SVI following SPA for four representative patients. The *x*-axis represents the time in seconds after SPA, and the *y*-axis shows the relative change in SVI compared to the baseline. Negative percentage values indicate an increase in SVI, while positive values represent a decrease. SPA = spinal Anesthesia, SVI = stroke volume index.

Following SPA, all patients were positioned in a lithotomy position with their legs placed in stirrups and a left lateral tilt of approximately 15°. The maximum sensory block height was achieved was at the T5 dermatome level. The median time to first occurrence of hypotension was 292 s [IQR 204–368]. Relative to baseline, the median SVI decreased significantly by 18.9% [IQR –6.1 – –31.5]; *p* < 0.001. At the time of first hypotension, 10 patients showed an increase in SVI from baseline, 12 patients had a decrease in SVI of 0 to 19.9%, and 29 patients had a decrease in SVI of 20% or more. Among these 29 patients, the median time from the onset of the SVI decline of 20% or more to the occurrence of hypotension was 2:20 min [IQR 1:16–3:56]. The percentage changes in the additional hemodynamic parameters are presented in Table 2 and Figures 2, 3. Dynamic changes in SVI following SPA for four representative patients are presented in Figure 4.

All the 51 patients received fractional intravenous boluses of C/T for MAP restoration. Dosages were reported based on the cafedrine component. The median cumulative cafedrine dose was 160 mg [IQR 120–200]. None of the patients had an estimated blood loss exceeding 500 mL. Data were collected at the end of surgery, with a median duration of 71 min [IQR 62–85].

### Hemodynamic response to vasopressor therapy

Following vasopressor administration, SBP increased by 26.8% [IQR 17.0–40.9]; *p* < 0.001. Total peripheral resistance index (TPRI) also rose by 30.4% [IQR 17.8–58.0]; *p* < 0.001. In contrast, HR decreased by 12.7% [IQR –27.8 – –2.8]; *p* < 0.001, while SVI increased by 10.9% [IQR –9.3 – 20.6]; *p* = 0.0623. All values were derived from paired measurements obtained immediately before vasopressor administration and after restoration of blood pressure, defined as SBP ≥ 80% of baseline. Additional hemodynamic parameters are presented in Table 3.

**Table 3:** Hemodynamic changes before and after blood pressure therapy.

Parameter	Before blood pressure therapy	After blood pressure recovery above the threshold	Percentage change	*P*-value
HR (bpm)	97 [80–113]	78 [71–99]	−12.7 [−27.8 – –2.8] %	*p* < 0.001
CI (l min^−1^ m^−2^)	3.4 [2.9–3.8]	3.1 [2.7–3.7]	−7.1 [−18.8 – 1.0] %	*p* = 0.0038
TPRI (dyn s ^−5^ m^−2^)	1,652 [1389–2,100]	2,297 [1829–2,753]	30.4 [17.8 – 58.0] %	*p* < 0.001
SVI (ml m^−2^)	34 [30–42]	37 [33–42]	10.9 [−9.3 – 20.6] %	*p* = 0.0623
SBP (mmHg)	100 [93–106]	126 [119–139]	26.8 [17.0 – 40.9] %	*p* < 0.001
CPI (W m^−2^)	0.5 [0.4–0.6]	0.6 [0.5–0.7]	7.9 [−10.0 – 35.6] %	*p* = 0.0789

Data are based on the treatment of 44 out of 51 patients, each contributing paired measurements before and after antihypotensive management. Seven patients who received their first vasopressor dose only after skin incision were excluded, as intra-abdominal surgical manipulation could have confounded the hemodynamic measurements. Data are reported as median values with interquartile range (IQR). Reported p-values reflect the statistical significance of paired within-subject comparisons between pre- and post-treatment values. CI = cardiac index, CPI = cardiac power index, HR = heart rate, SBP = systolic blood pressure, SVI = stroke volume index, TPRI = total peripheral resistance index.

### Analysis of signal variability and accuracy

The total observation time for the 51 analyzed patients was 3,781 min. With a 4-s measurement interval, this corresponds to a theoretical maximum of 56,713 data points per parameter. Due to intermittent signal acquisition errors, 257 data points were missing for both CI and SVI, equating to approximately 4.08 missing values per hour of observation (0.45%). For HR, 117 values were missing, corresponding to approximately 1.86 missing values per hour (0.21%).

The available dataset was used to assess the agreement between the original 4-s measurements and the simulated 20-s intervals. The CI showed a MAPE of 2.27% and a RMSE of 0.09 L/min/m^2^. The SVI showed a MAPE of 2.20% and an RMSE of 1.01 mL/m^2^. The HR showed a MAPE of 0.90% and an RMSE of 0.93 bpm (Figure 4).

## Discussion

This observational study investigated hemodynamic responses in 51 women who developed maternal hypotension following SPA for CD, using continuous non-invasive BR monitoring. Hypotension typically occurred within minutes of neuraxial blockade and was characterized by a gradual, rather than abrupt, decline in arterial pressure. Among the hemodynamic parameters assessed, SVI demonstrated one of the most pronounced and statistically significant reductions in most participants. In 29 patients, a sustained SVI reduction of ≥ 20% from baseline preceded the first hypotensive episode. The median time interval between the onset of this decline and the occurrence of hypotension was just over 2 min. Despite the high temporal resolution of 4-s measurements, data loss due to motion artifacts was minimal and clinically negligible. This suggests a potential methodological advantage of short-interval monitoring over conventional 20-s averaging, particularly when evaluating hemodynamic variability following SPA.

In clinical practice, both absolute and relative changes in BP are commonly used to guide hemodynamic therapy (3). However, the optimal timing for baseline BP measurement remains under investigation (28). In our study, most hemodynamic parameters differed between the ward and OR. While SVI remained relatively stable, HR, SBP, and CI increased in the OR. These differences may influence the interpretation of the relative SBP changes when managing maternal hypotension. Using an inadequate baseline could result in unnecessary vasopressor administration and overshooting hypertension (28). The observed stability of SVI in our study suggests that including SVI – alongside BP – may enhance the interpretation of relative hemodynamic changes and improve management strategies, particularly in high-risk obstetric population. However, further studies are required to validate this approach.

Given the significant decrease in SVI observed after SPA in our study, this finding highlights the potential utility of continuous SVI trend monitoring as a surrogate parameter to support more individualized hemodynamic management.

In more than half of our participants, a ≥ 20% reduction in SVI was observed following SPA, which is a threshold generally regarded as clinically relevant. Notably, hypotension did not occur immediately but was typically delayed by more than 2 minutes. In such cases, the marked decline in SVI may serve as an early indicator of impending hemodynamic compromise, even though blood pressure measurements - taken at one-minute intervals - remained within the normotensive range during this phase. In clinical settings that favor the prophylactic management of hypotension, the interpretation of SVI trends could aid in guiding the adjustment of vasopressor infusion rates. Furthermore, other groups have already investigated the prediction of hypotension using non-invasive hemodynamic monitoring (3, 12, 29). However, further studies are necessary to establish robust prediction models for hypotension during CD (30), and our findings may help guide such efforts.

The impact of maternal positioning after spinal anesthesia on blood pressure and SVI is widely discussed, particularly because supine positioning may cause inferior vena cava compression and reduce venous return (31). Preoperative supine SVI values after rest were highly comparable to those recorded in the OR before spinal anesthesia. Accordingly, the subsequent changes observed after spinal anesthesia are most likely attributable to sympathetic blockade rather than to inferior vena cava compression. Although the available literature remains somewhat inconsistent, recent bioreactance data demonstrated no significant differences in cardiac output, stroke volume, or blood pressure between supine and tilted positions (32).

In our cohort, restoration of blood pressure was primarily associated with a significant increase in systemic vascular resistance, most likely reflecting the well-known *α*-adrenergic vasoconstrictive effect mediated by the norepinephrine component of theodrenaline. These findings are broadly consistent with results from previous studies (23, 26), which may indicate reliable performance of bioreactance technology in this setting. At the same time, HR showed a marked decrease, returning closer to the preoperative ward values. This effect may represent a compensatory mechanism related to BP stabilization and vasopressor-induced vasoconstriction, whereas SVI demonstrated only a modest, non-significant rise of 11%. However, the primary aim of this study was to assess the feasibility of bioreactance-based hemodynamic monitoring. The study protocol included a standardized observation period up to the first hypotensive episode, whereas the treatment of hypotension was left to the discretion of the attending anesthesiologist and was not standardized. In addition, seven patients who received their first vasopressor dose only after skin incision were excluded, as intra-abdominal surgical manipulation could have confounded the hemodynamic measurements. These factors restrict the generalizability of the findings on the hemodynamic response to vasopressor therapy.

The extent to which maternal hypotension affects the fetus remains unclear. The duration of maternal hypotension may be more important than the absolute decrease (4, 33). This is supported by our data, as timely treatment of hypotensive events in our study was associated with uniformly unremarkable neonatal outcomes, with Apgar scores and umbilical arterial blood gases within normal reference ranges (34). These findings indicate that both the hypotensive episodes observed after SPA and the associated changes in advanced hemodynamic parameters, particularly the decline in the SVI, did not translate into alterations in acid–base status. Thus, no clinically relevant neonatal compromise was observed in the low-risk population. However, we did not assess uteroplacental perfusion indices, which could provide further insight into fetal oxygenation (35, 36). The unremarkable neonatal values in our cohort should be interpreted in the context of elective cesarean delivery in healthy women. Accordingly, the generalizability of these findings to high-risk scenarios remains limited, underscoring the need for targeted studies in such populations. In this context, it also remains uncertain whether early stabilization of the SV following SPA during CD leads to a reduction in the incidence or severity of intraoperative hypotension. However, this strategy - particularly when leveraging high-resolution monitoring at 4-s intervals - could be of interest for further research.

Many studies employing advanced hemodynamic monitoring rely on systems that generate averaged values at 20-s intervals. To our knowledge, this is the first study to report the use of the Starling™ monitor (Baxter, USA) for continuous hemodynamic monitoring during CD under SPA at 4-s intervals. This high-resolution sampling frequency enables the acquisition of five times more data points compared to conventional 20-s averaging, potentially allowing for a more detailed and dynamic analysis of HR, SVI, and CI trends. The low MAPE and RMSE values observed for HR, SVI, and CI indicate a high level of agreement between the raw 4-s data and the corresponding simulated 20-s averages, suggesting that the 4-s measurements were both stable and robust. From a clinical perspective, the RMSE values remained within a range unlikely to influence therapeutic decisions. These findings support the feasibility, accuracy and potential utility of high-resolution data acquisition using 4-s intervals. However, further research is required to determine whether this increased temporal resolution translates into improved clinical outcomes. Depending on specific research or clinical context, 20-s measurement intervals may still be appropriate.

Previous validation studies have consistently shown that BR provides reliable hemodynamic measurements in pregnancy, particularly in the third trimester, and that its accuracy is not substantially influenced by maternal characteristics (10, 37–39). Although some studies suggest that body size, ethnicity, or maternal cardiac disease may influence absolute values, the interpretation of hemodynamic trends rather than absolute measurements appears appropriate (40, 41). Taken together, these findings support the validity of BR in obstetric populations and strengthen the rationale for our study approach using high-resolution trend analysis with a 4-s interval.

In addition to the BR technology evaluated in this study, finger cuff systems are a well-established alternative for continuous non-invasive hemodynamic monitoring (7, 42, 43). These devices offer not only continuous BP measurements but also derived advanced hemodynamic parameters. Despite their widespread use, several studies have reported that the accuracy of finger cuff-based measurements may fall outside clinically acceptable tolerance limits (6–8). To date, no direct comparison between BR and finger cuff technologies has been published, highlighting a relevant gap for future investigations.

### Strength and limitations

This study is the first to report the application of the Starling™ monitor (Baxter, USA) for continuous hemodynamic assessment at 4-s intervals during SPA for CD. The measurements were found to be stable and robust, offering five times the data density of conventional 20-s averaging and allowing for a more detailed analysis of HR, SVI, and CI trends. Nonetheless, this study has several limitations that must be acknowledged.

First, although multiple international and national guidelines address obstetric anesthesia and the management of hypotension during CD (2, 3), our institutional protocol adhered to current national recommendations, which advocate a therapeutic rather than a prophylactic approach to hypotension management (16). These recommendations are currently being revised. Although existing evidence supports the use of prophylactic vasopressors, variations in guidelines reflects broader differences in clinical practice and healthcare systems across regions.

Second, this study was not designed to compare patients who experienced hypotension with those who did not.

Third, the neonatal assessment was limited to Apgar scores and umbilical arterial blood gas levels. We did not obtain maternal lactate values or uteroplacental perfusion, which would have provided additional insights into maternal-fetal oxygenation and neonatal metabolic status. This limits the ability to draw conclusions regarding the clinical consequences of the observed hemodynamic changes. As this single-center study was restricted to healthy women undergoing elective CD, the generalizability of our findings is limited. In addition, no data were collected on emergency procedures or high-risk obstetric scenarios, including preeclampsia, placenta accreta spectrum, or major hemorrhage.

Fourth, SVR values were calculated without central venous pressure measurement using the simplified formula 80 × (MAP-CVP)/CI, as no central venous catheter was placed.

Fifth, although the BR method has shown good agreement with echocardiography for CO measurement in pregnant women (10, 11), current evidence remains insufficient and somewhat inconsistent to support its routine use in obstetric anesthesia (38, 41, 44).

Sixth, BP was not measured continuously but at intervals of 1 min. If hypotension occurred shortly after a significant drop in SVI, it might not have been detected until the next scheduled measurement. This limitation reduces the accuracy of determining the actual latency between the onset of SVI decline and hypotension. Continuous arterial blood pressure monitoring would allow for a more accurate temporal correlation and better assessment of the early warning potential of SVI trends.

Seventh, another important limitation was the absence of a comparator arm. Without such a control, the relative benefits of bioreactance monitoring cannot be directly assessed. Predefined interventions triggered by a relevant decline in the SVI (≥20% or a sustained downward trend) could have moved BR monitoring beyond feasibility toward clinical applicability with measurable effects on hypotension and neonatal outcomes. This limitation should be acknowledged, and future studies should be designed accordingly.

Eighth, vasopressor therapy was not standardized and seven patients treated only after skin incision were excluded, limiting comparability and generalizability of the hemodynamic response analysis.

Despite these limitations, the present study provides novel, valuable insights into the hemodynamic changes following SPA for parturients, using high-resolution BR-based methods. These findings contribute to the understanding of maternal cardiovascular dynamics during CD and offer a methodological foundation for future research in high-risk obstetric populations.

## Conclusion

This hypothesis-generating, prospective observational study is the first to investigate the feasibility of continuous hemodynamic monitoring using a BR-based system with a 4-s measurement interval during CD under SPA. The data obtained were stable and robust, offering five times the resolution of conventional 20-s averaging and enabling a more detailed analysis of HR, SVI, and CI trends. Among the parameters assessed, SVI demonstrated the highest consistency between the preoperative (ward) and intraoperative (OR) settings, supporting its potential role as a reliable reference for defining hemodynamic baseline. Notably, a reduction in SVI ≥ 20% at the time of the first hypotensive episode was observed in more than half of the patients, suggesting that relative SVI changes may serve as an early surrogate parameter of impending maternal hypotension, even before overt declines in BP are detected.

The findings from this low-risk obstetric cohort provide a methodological basis for future investigations and may support the application of BR monitoring in more complex or high-risk obstetric populations.

## Data Availability

The raw data supporting the conclusions of this article will be made available by the authors upon reasonable request, subject to approval by the corresponding author and in accordance with institutional and ethical regulations.

## Glossary

ASA: American Society of Anesthesiologists classification
BMI: Body mass index
BP: Blood pressure
BR: Bioreactance
C/T: Cafedrine/theodrenaline
CI: Cardiac index
CPI: Cardiac power index
CD: Cesarean delivery
GA: General anesthesia
HR: Heart rate
IONV: Intraoperative nausea and vomiting
IOH: Intraoperative hypotension
LA: Local anesthesia
MAP: Mean arterial pressure
MAPE: Mean absolute percentage error
NIBP: Non-invasive blood pressure
OR: Operating room
RMSE: Root mean square error
SBP: Systolic blood pressure
SpO_2_: Peripheral oxygen saturation
SPA: Spinal anesthesia
SV: Stroke volume
SVI: Stroke volume index
TPRI: Total peripheral resistance index
